# Direct Cortical Motor Evoked Potentials Versus Transcranial Motor Evoked Potentials for the Detection of Cortical Ischemia During Supratentorial Craniotomy: Case Report

**DOI:** 10.7759/cureus.3771

**Published:** 2018-12-24

**Authors:** Justin W Silverstein, Andrew Rosenthal, Jason A Ellis

**Affiliations:** 1 Neurology, Neuro Protective Solutions, Brooklyn, USA; 2 Neurosurgery, Lenox Hill Hospital Northwell Health, New York, USA

**Keywords:** aneurysm clipping, craniotomy, neurosurgery, neuromonitoring, neurophysiological monitoring, direct cortical stimulation, intraoperative monitoring, aneurysm, motor-evoked potentials, direct cortical motor-evoked potentials

## Abstract

Transcranial motor evoked potential (TCMEP) and direct cortical motor evoked potential (DCMEP) paradigms have historically been used contemporaneously or independently for supratentorial craniotomies. DCMEP provides focal stimulation to the cortical surface, whereas TCMEP stimulation is more variable and may be activating structures deeper than those at risk during a supratentorial craniotomy.

We present the case report for a 65-year-old female who underwent a supratentorial craniotomy for the clipping of a right-sided unruptured middle cerebral artery (MCA) aneurysm. DCMEP recordings of the upper extremity degraded after the parent vessel was temporarily occluded with a clip. The recordings returned once the clip was released. The DCMEP lower extremity recordings did not deviate from their established baseline. TCMEP recordings (upper and lower extremities) also did not deviate from their established baselines. The permanent clip was placed without incident, and the patient awoke neurologically intact.

This case study demonstrates the specificity and sensitivity of DCMEP vs. TCMEP. DCMEP activates the corticospinal tract more superficially; therefore, it was evident by the loss of the upper extremity DCMEPs without the loss of lower extremity DCMEPs that the temporary vessel occlusion caused an ischemic event focal to the cortical area perfused by the MCA. This ischemic event was not detected by TCMEP.

## Introduction

Introduced by Fritsch and Hitzig, direct stimulation to evoke eloquent areas of the cerebral cortices has been used in neurosurgical procedures since the late 19th century [1-2]. In the 1930s, Penfield and Boldrey standardized the mapping of eloquent areas of the brain using a long train bipolar stimulation method [2-4]. The Penfield method consists of stimulating the exposed cortex in awake patients for 4 to 5 seconds using a bipolar stimulating probe with a long train of 60 Hz or 50 Hz [3]. With the advent of multi-pulse stimulation, Taniguchi et al. [5] applied short train anodal stimulation directly to the exposed motor cortex while patients were under general anesthesia and elicited compound muscle action potentials (CMAP) from select target muscles where EMG needles were placed. Short train multi-pulse stimulation allows for instantaneous feedback from the motor system compared to long train stimulation. The risk of electrically-induced seizure is also reduced using short train multi-pulse stimulation [3].

Other investigators expanded upon Taniguchi’s application of short pulse-trains to elicit motor evoked potentials (MEP) using transcranial stimulation in the late 1990s [6-8]. The ability to use transcranial stimulation made transcranial motor evoked potentials (TCMEPs) applicable to any procedure where the corticospinal tracts are at risk. Currently, many different surgical procedures use TCMEP monitoring, including supratentorial craniotomy. TCMEP and direct cortical motor evoked potential (DCMEP) paradigms have historically been used contemporaneously or independently during supratentorial craniotomy. The use of DCMEPs for monitoring the corticospinal tracts during craniotomy has been well described [5, 9-12]. DCMEP has many advantages over TCMEP monitoring. For example, there is limited-to-no patient movement; therefore, the stimulation can be conducted continuously throughout a procedure allowing for real-time feedback of motor system integrity. DCMEP also allows for a more focal superficial stimulation. Using a single contact from a strip or grid electrode placed directly on the exposed cortex allows for the use of comparatively lower stimulation intensity, therefore, reducing current density, current spread, and deep penetration of stimuli [3].

When using TCMEP during supratentorial craniotomy, the literature suggests that it is paramount not to elicit recordings from the limbs ipsilateral to the cortical site of activation, colloquially known as the “crossover” response [13-16]. When the "crossover" response occurs, the activation site from TCMEP stimulation is thought to be subcortical [17-18]. The deeper penetration of stimulus is due to the amount of current intensity used, current density, and current spread that occurs while applying transcranial stimulation. In theory, the cortical structures at risk are being bypassed by the transcranial stimulation when the "crossover" response is present and false negatives may occur. For this reason, the “crossover” response has become the marker for discriminating the activation of subcortical sites vs. cortical sites. However, there is very little empirical support to suggest that when the “crossover” response is not present, the cortical area is still not being bypassed with transcranial stimulation. Therefore, precluding the “crossover” response does not necessarily indicate that the current delivered is not penetrating deeper than the structures at risk. The authors of this paper intend to illustrate a craniotomy for the clipping of an unruptured middle cerebral artery aneurysm where direct cortical motor evoked potentials were sensitive and specific to vascular insult, whereas transcranial motor evoked potentials were not.

## Case presentation

**Figure 1:**
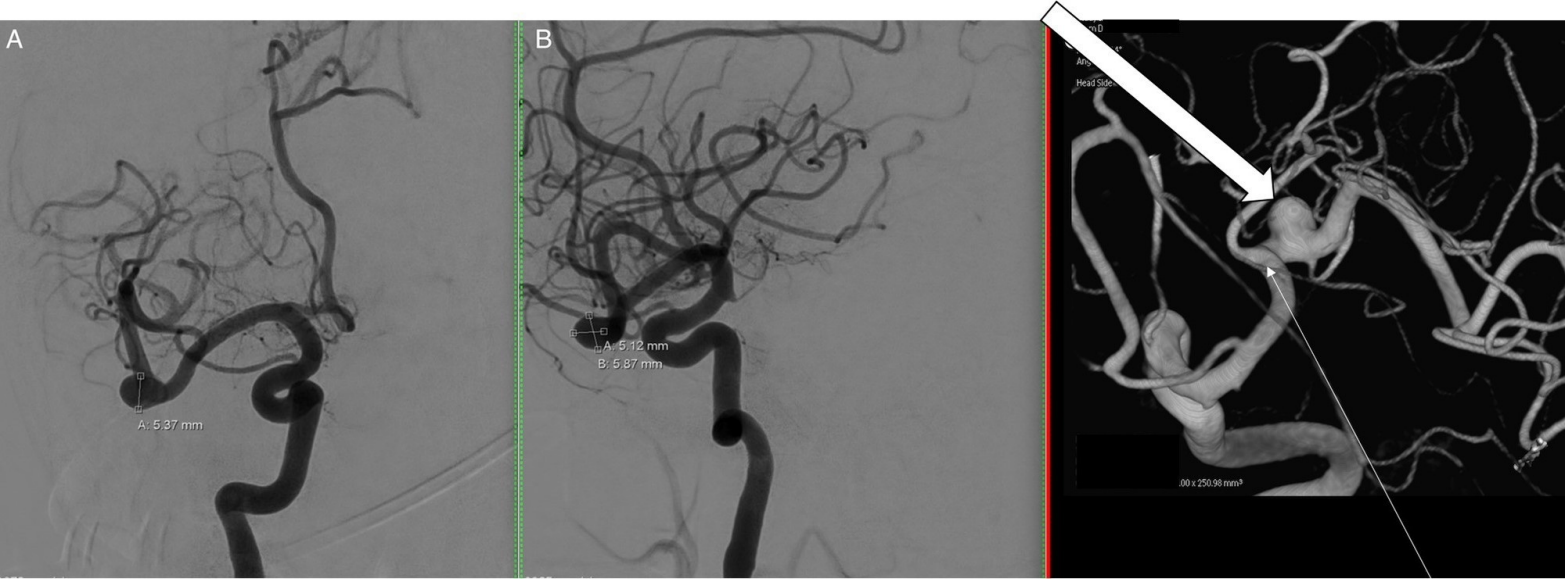
Right internal carotid arteriogram Right internal carotid arteriogram in anteroposterior (AP) (A), lateral (B), and 3-dimensional (C) views demonstrate a nearly 6 mm saccular aneurysm (block arrow) arising just distal to the middle cerebral artery bifurcation (thin arrow).

A 65-year-old female presented to the Department of Neurosurgery with an unruptured right middle cerebral artery (MCA) aneurysm (Figure 1). Other than a left facial droop and subsequent left hemifacial spasm (which is incidental to an aneurysm), the patient had no relevant past medical or surgical history. The patient was admitted to the hospital for a right-sided craniotomy for clipping of a right MCA aneurysm. A pterional craniotomy was performed under general anesthesia. 

Intraoperative neurophysiological monitoring methods

Median, ulnar, and posterior tibial nerve somatosensory evoked potentials (SSEP) were used in standard fashion. TCMEP and DCMEP were used for functional monitoring of the motor pathways. For TCMEP, a multi-pulse train of six was used with a pulse width of 50 µsec and an interstimulus interval (ISI) of 2 ms (500 Hz). Voltage stimulation between 100 and 300 volts was applied (controlling for the inhibition of the “crossover” response). Stimulation parameters for DCMEP included a multi-pulse train of five delivered at a constant current of 15 mA with a pulse width of 500 µsec and an ISI of 2 ms (500 Hz). Upper extremity and lower extremity muscles were used as target acquisition sites using standard intraoperative electromyography (EMG) needle electrodes for TCMEP and DCMEP.

Transcranial motor evoked potential methods

**Figure 2:**
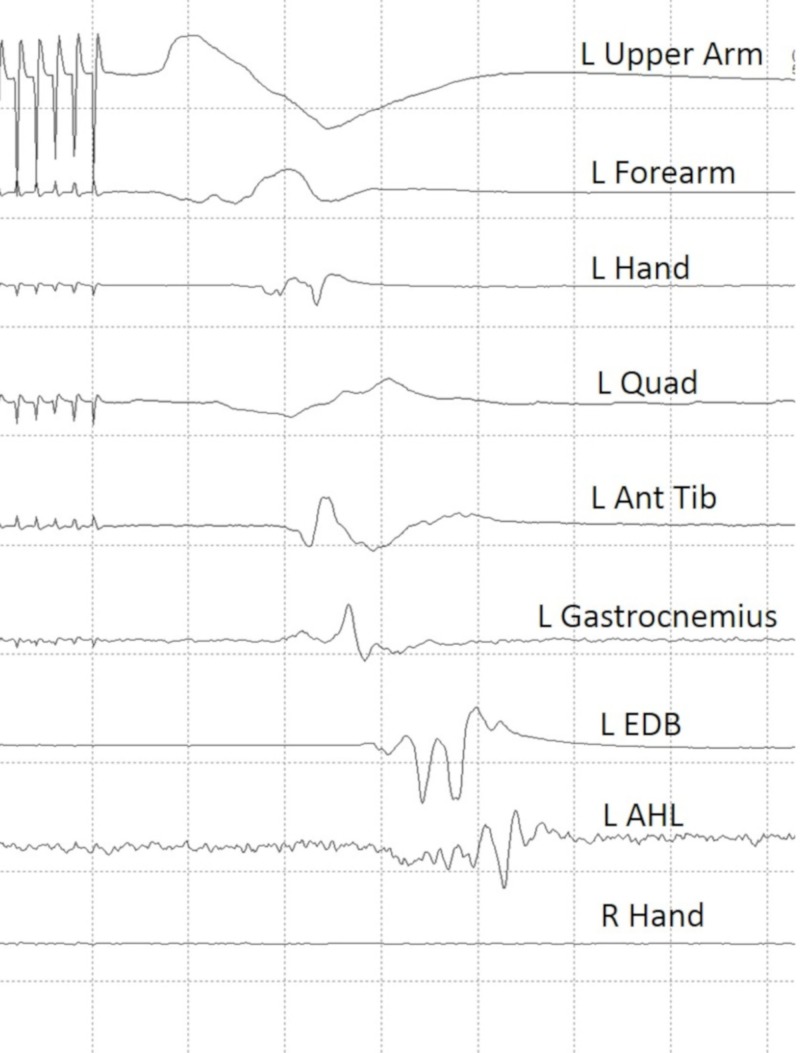
Baseline transcranial motor evoked potential data Established preincision baseline transcranial motor evoked potential data. Note that there is no response elicited from the right hand indicating that there is no “crossover” response. L upper arm: left biceps/triceps muscles; L forearm: left extensor digitorum communis/flexor carpi radialis muscles; L hand : left abductor pollicis/left abductor digiti minimi; L quad: left quadriceps muscle; L ant tib: left anterior tibialis muscle; L EDB: left extensor digitorum brevis; L AHL: left abductor hallucis longus; R hand: right abductor pollicis brevis/right abductor digiti minimi.

TCMEP data was collected before incision. The stimulus was delivered and optimized to inhibit a “crossover” recording from the ipsilateral hand muscles. Three sets of stimulation electrodes were affixed to the patient’s scalp using corkscrew needle electrodes, providing three channels of motor stimulation to switch between using a TCS 4 constant voltage stimulator unit (Cadwell, Kennewick, WA). This technique (involving the use of multiple stimulation channels) allowed for better optimization of the TCMEP recordings (Figure 2). Scalp electrodes for TCMEP stimulation were placed so as not to disturb the surgical site.

Direct cortical motor evoked potential methods

**Figure 3:**
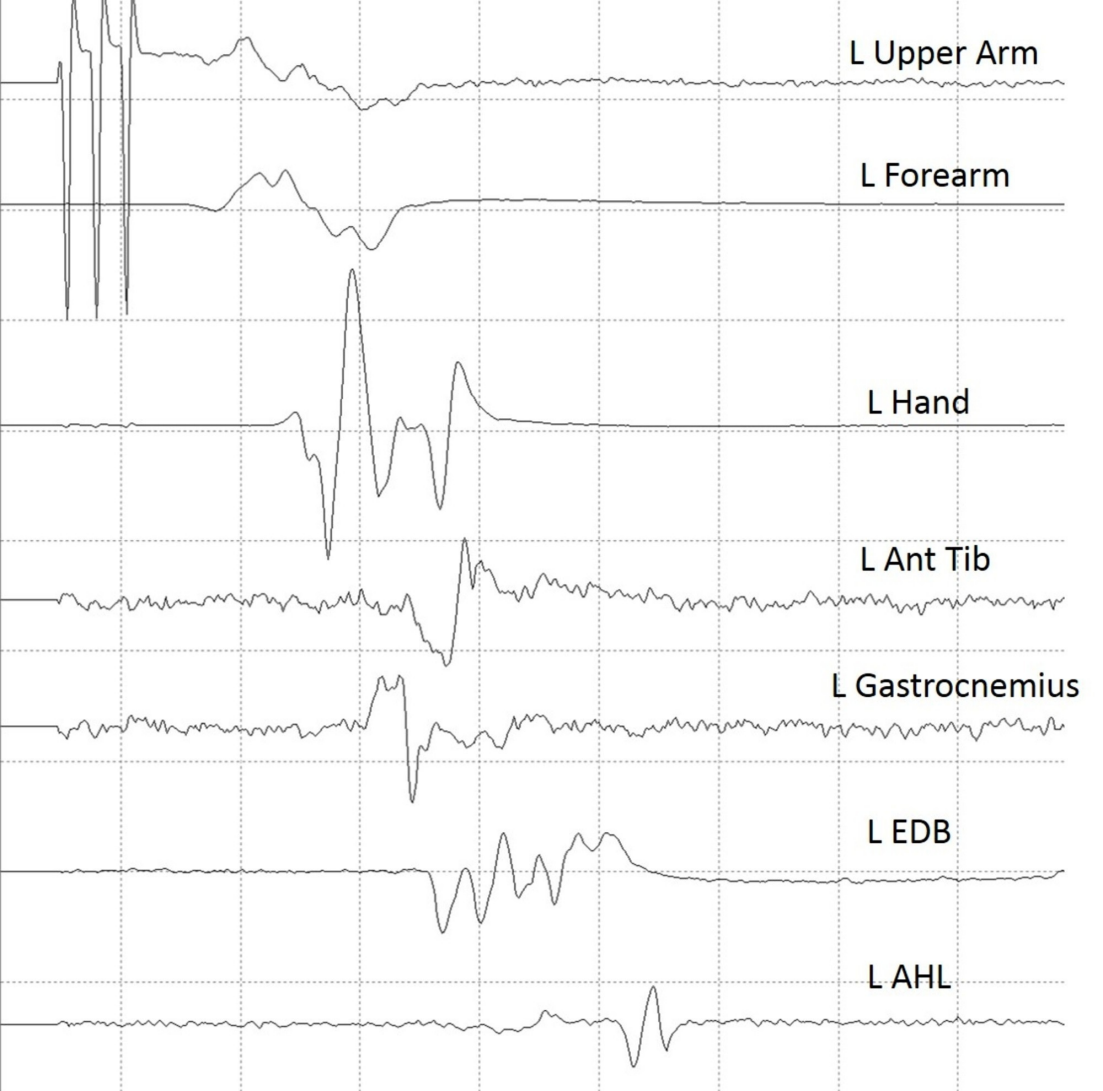
Established baseline data for direct cortical motor evoked potentials Established baseline data for direct cortical motor evoked potentials using contact number 2 from a 1 x 6 strip electrode. L Upper arm: left biceps/triceps muscles; L Forearm: left extensor digitorum communis/flexor carpi radialis muscles; L Hand: left abductor pollicis/left abductor digiti minimi; L Ant Tib: left anterior tibialis muscle; L Gastroc: left gastrocnemius muscle; L EDB: left extensor digitorum brevis; L AHL: left abductor hallucis longus

Once the dura mater was open and the aneurysm located, a 1 x 6 strip electrode was slid under the dura and skull towards the precentral gyrus for DCMEP stimulation. Before DC stimulation was initiated, the left median nerve was stimulated at 25 mA, and somatosensory evoked responses were recorded directly from the cortical surface to ensure that the strip electrode was adequately placed over the motor area. All six contacts from the strip electrode demonstrated adequate placement over the motor cortex. Next, all six contacts from the strip electrode were plugged into six different positive output channels of an ES-IX constant current stimulator unit (Cadwell, Kennewick, WA) to allow for anodal stimulation. Six off-site returns (corkscrew electrodes placed on the scalp) were used as cathodes (negative) for each stimulation channel. Using multiple stimulation channels allowed the neurophysiologist to switch between each electrode contact from the strip electrode to find optimal DCMEP recordings. Stimulation at contact 2 from the strip electrode yielded the most reliable and robust DCMEP recordings from the targeted upper and lower extremity muscle groups (Figure 3).

**Figure 4:**
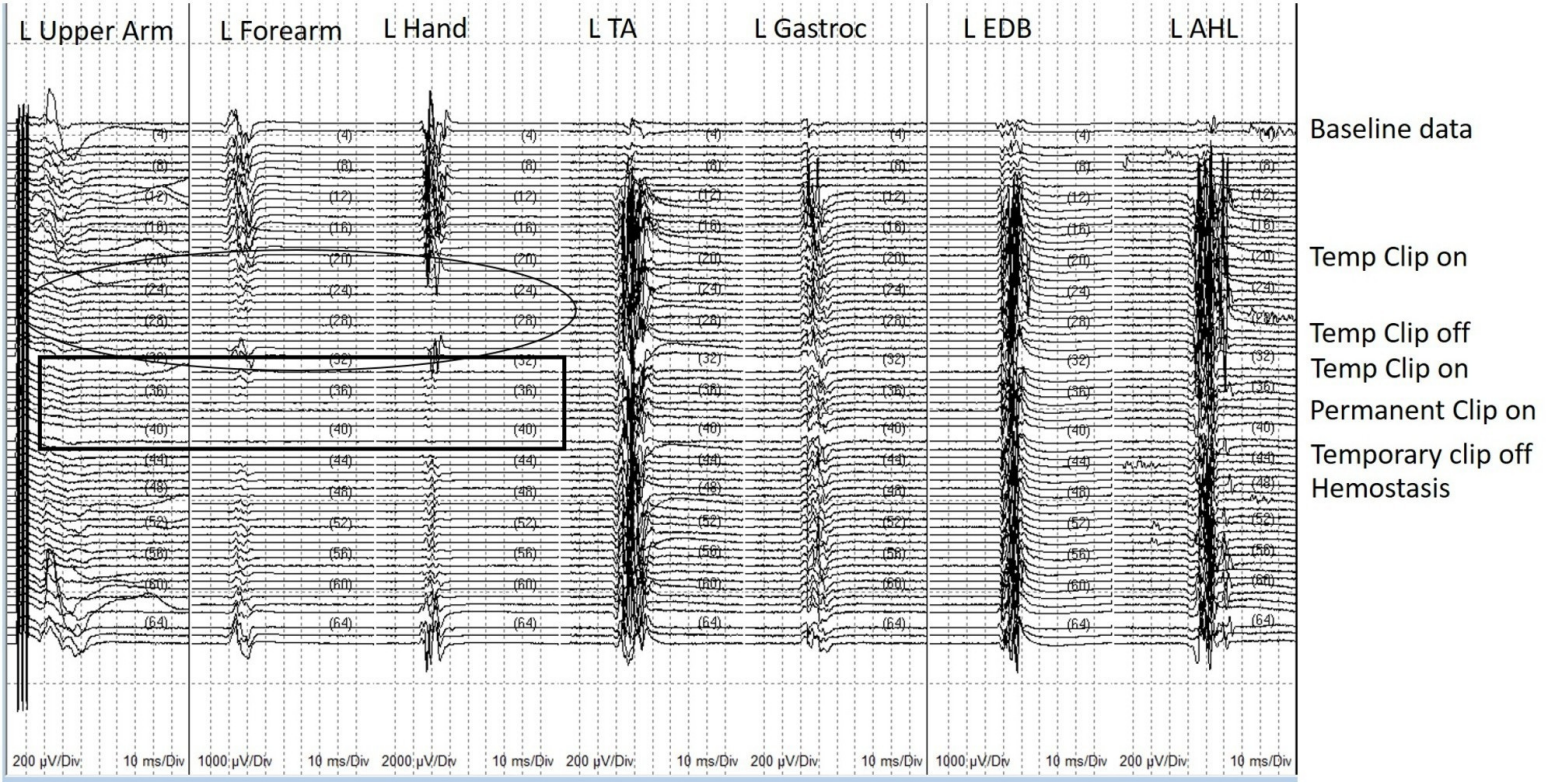
Direct cortical motor evoked potential (DCMEP) recordings for the entire procedure in chronological order DCMEP recordings for the entire procedure in chronological order from the establishment of baselines (top) to closing (bottom). Note the deterioration of upper extremity muscles when temporary vessel occlusion of the parent artery occurs. Also, note that the recordings returned each time the temporary vessel occlusion was released. The circle indicates the first attenuation of DCMEP recordings with subsequent return after temporary occlusion is released. The rectangle indicates loss of DCMEP upper extremity muscles after the temporary occlusion for a second time. L upper arm: left biceps/triceps muscles; L forearm: left extensor digitorum communis/flexor carpi radialis muscles; L hand: left abductor pollicis/left abductor digiti minimi; L Ant Tib: left anterior tibialis muscle; L Gastroc: left gastrocnemius muscle; L EDB: left extensor digitorum brevis; L AHL: left abductor hallucis longus

**Figure 5:**
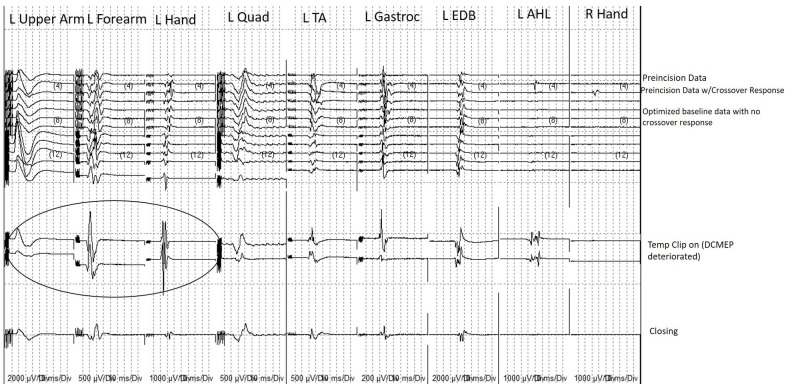
Transcranial motor evoked potential (TCMEP) recordings for the entire procedure in chronological order TCMEP recordings for the entire procedure in chronological order from the establishment of pre-incision baseline data (top) to closing data (bottom). Note the crossover response present as the neurophysiologist worked on optimizing the recordings. Note that the 1) crossover response was inhibited prior to the start of the procedure and 2) the TCMEPs did not deteriorate or deviate from their established baselines at the time when the DCMEPs deteriorated due to temporary vessel occlusion (Circle). L Upper arm: left biceps/triceps muscles; L Forearm: left extensor digitorum communis/flexor carpi radialis muscles; L Hand: left abductor pollicis/left abductor digiti minimi; L Quad: left quadriceps muscle; L Ant Tib: left anterior tibialis muscle; L Gastroc: left gastrocnemius muscle; L EDB: left extensor digitorum brevis; L AHL: left abductor hallucis longus; R Hand: right abductor pollicis brevis/right abductor digiti minimi.

Once reliable and reproducible recordings from DC stimulation were obtained, the DCMEP was prioritized over other neuromonitoring modalities for the remainder of the procedure. The DCMEP recordings attenuated from all upper extremity muscle groups during the temporary occlusion of the parent vessel. The lower extremity DCMEP recordings did not deviate from their established baseline. At this point, the surgeon removed the temporary clip which yielded an immediate return of the upper extremity DCMEP recordings. Once blood flow was restored to the MCA and maintained, temporary occlusion of the parent vessel was performed for a second time. Again, the DCMEP upper extremity recordings attenuated in amplitude and were eventually not obtainable (Figure 4). At this point, TCMEP stimulation was conducted and there was no diminution noted compared to the baseline recordings (Figure 5). All upper extremity TCMEP recordings remained within established baseline limits, whereas the DCMEP upper extremity recordings were absent. The permanent clip was placed across the neck of the aneurysm and the temporary clip was immediately removed. The DCMEP upper extremity recordings returned almost immediately after the temporary vessel occlusion was released, and the signals remained within baseline limits for the remainder of the procedure. Hemostasis was achieved, and closure was performed in standard fashion. The patient awoke neurologically intact. SSEPs were not conducted during the time of the DCMEP deterioration, which is a limitation to the case study.

## Discussion

Our illustrative case discusses the specificity and sensitivity of DCMEP vs. TCMEP without a “crossover” response. DCMEP activates the corticospinal tract more superficially; therefore, it is evident by the loss of upper extremity DCMEPs without the loss of lower extremity DCMEPs that temporary vessel occlusion of the parent artery was creating an ischemic event focal to the cortical area perfused by the MCA which was not detected by the TCMEP.

It is suggested that when conducting TCMEPs during supratentorial craniotomy the neurophysiologist controls for the “crossover” response. This is based on the hypothesis that if a “crossover” response occurs, then the activation of the motor tracts may be occurring deeper than the structures at risk. Subcortical activation was described by Edgley et al. [18], where they posit that scalp stimulation at higher intensities activates the corticospinal tracts in the pons or the pyramids instead of the cortex. Burke et al. [17] discussed how initially lower intensity scalp stimulation activated cortical neurons within the cortex; however, as stimulus intensity was increased, the activation shifted to deeper subcortical structures. Though this is true and makes sense physiologically, there is no stimulus intensity described that can be used to discriminate between exciting superficial cortical areas vs. deeper structures with transcranial stimulation. Current density, current spread, and depolarization of the motor neuron pool to transcranial stimulation are multifactorial and variable from patient-to-patient, especially after considering effects of anesthesia, hemodynamics, underlying co-morbidities, and preoperative weakness [19-20]. It can be inferred that if a “crossover” recording is obtained, the stimulation is likely penetrating deeper than the cortex. However, there are times when the “crossover” response is not obtained, and yet, the stimulation is still penetrating deeper than the cortical area, as shown in our case. Therefore, if the neurophysiologist relies solely on the paradigm of not obtaining a “crossover” recording as a means to monitor the function of the motor cortex with TCMEP, then they are providing a false sense of security to the surgeon that may lead to a higher false-negative rate and increased postoperative sequelae.

Motoyama et al. [15] had similar findings when they conducted both DCMEP and TCMEP on 48 patients with unruptured aneurysms. They found that when the MCA was occluded, the DCMEP recordings diminished but the TCMEP recordings did not. They also controlled for the “crossover” response and did not obtain “crossover” responses during the cases where the TCMEP remained stable in the face of a degraded or lost DCMEP. Lee et al. [16] described the sensitivity and specificity of TCMEP in spinal surgery as 100% and 83%, respectively; however, they went on to describe the sensitivity in supratentorial brain tumor operations of TCMEP to be 75% and the specificity to be 77.8%. They indicated in their research that the lower sensitivity and specificity for craniotomy vs. spine surgery was due to TCMEP stimulation activating deep white matter, which has a limited effect in spinal surgery but can create false-negatives when applied during craniotomy [16]. Li et al. [14] also found that they had preservation of the TCMEP with loss of the DCMEP and SSEP, indicating that the TCMEP did not detect cortical ischemia. Their patient sustained an intraoperative MCA stroke during resection of a supratentorial tumor and subsequently awoke from anesthesia hemiplegic.

## Conclusions

TCMEP and DCMEP are part of the neurophysiologist’s and neurosurgeon’s armamentarium and should be used accordingly. DCMEPs are shown to be more specific and more sensitive than TCMEPs in supratentorial craniotomy and should be employed when feasible. TCMEPs should not be discarded or discontinued, as they do have benefits in cranial surgery. For example, the establishment of pre-incision baseline data can be used as a pilot to what should be expected from DC stimulation. However, the expectations of their utility as an indicator of the cortical function should be managed, and if only using TCMEP, it should be understood that the risk of a false-negative occurring is increased, even if the neurophysiologist has controlled for the “crossover” response.
